# The anti-*Candida* activity by Ancillary Proteins of an *Enterococcus faecium* strain

**DOI:** 10.3389/fmicb.2015.00339

**Published:** 2015-05-08

**Authors:** Utpal Roy, Ajay G. Chalasani, M. Raeesh Shekh

**Affiliations:** Department of Biological Sciences, Birla Institute of Technology and Science Pilani KK Birla Goa CampusVasco Da Gama, India

**Keywords:** antimycotic peptides, anti-*Candida* protein, *Enterococcus faecium*/lactic acid bacteria (LAB), Lysin M, *Candida albicans*

## Abstract

An antimycotic activity toward seven strains of *Candida albicans* was demonstrated erstwhile by a wild-type *Enterococcus faecium* isolated from a penguin rookery of the Antarctic region. In the present study the antimicrobial principle was purified by ion exchange and gel permeation chromatography and further was analyzed by LC-ESI-MS/MS. In the purification steps, the dialyzed concentrate and ion exchange fractions inhibited *C. albicans* MTCC 3958, 183, and SC 5314. However, the gel filtration purified fractions inhibited MTCC 3958 and 183. The data obtained from the LC-ESI-MS/MS indicate that the antimicrobial activity of the anti-*Candida* protein produced by *E. faecium* is facilitated by Sag A/Bb for the binding of the indicator organism's cell membrane. Partial N-terminal sequence revealed 12 N-terminal amino acid residues and its analysis shown that it belongs to the LysM motif. The nucleotide sequence of PCR-amplified product could detect 574 nucleotides of the LysM gene responsible for binding to chitin of the cell wall of *Candida* sp.

## Introduction

Antimicrobial and antimycotic peptides are small cationic and amphipathic molecules, generally with fewer than 50 amino acids. These peptides are omnipresent and have been isolated from prokaryotes and eukaryotes in plants, bacteria, fungi, and animals (Zasloff, 2002; Bulet et al., 2004).

Amongst Lactic Acid Bacteria (LAB), members of the genus *Enterococcus* are widely distributed throughout nature as inhabitants of the gastrointestinal tract of humans and other animals and are also present in vegetables, plant materials and foods (Giraffa, 2002). Many LAB bacteriocins, particularly those produced by enterococci (enterocins), are characterized by their broad range of activity against many gram-positive bacteria (van Belkum and Stiles, 1995; Nes et al., 2007). Bacteriocins may also play an important role in maintaining bacterial community structures (Riley and Wertz, 2002) in specific ecological niche. They have also been proposed as probiotics for both the gastrointestinal and urogenital tracts (Redondo-Lopez et al., 1990). One of the main mechanisms used by the LAB to interfere with the colonization of pathogens and avoid proliferation of those potential pathogens is production of antimicrobial agents, such as organic acids, hydrogen peroxide and bacteriocins or related substances (Redondo-Lopez et al., 1990; Jack et al., 1995).

Though the vast majority of enterocins produced by *Enterococcus faecalis* and *Enterococcus faecium* are found active only against Gram-positive bacteria (Larsen, 1993), some exceptions with broad activity spectra have been described in recent years to show the ability of bacteriocins to inhibit the Gram-negative microorganisms (De Kwaadsteniet et al., 2005; Line et al., 2008). Reports on antifungal prowess of *E. faecium* or *E. faecalis* are relatively rare. It was demonstrated in one of the previous investigations that the antifungal compounds such as phenyllactic acid and 4-hydrophenyllactic acid were produced by *Lactobacillus plantarum* (Lavermicocca et al., 2000). Besides this, bacteriocin-like substances and other compounds were produced by *L. pentosus*, *L. coryniformis*, and *E. faecalis* (Okkers et al., 1999; Magnusson and Schnurer, 2001; Lupetti et al., 2002).

*Candida albicans* is the major human fungal pathogen of immunocompromised patients (Lupetti et al., 2002). The nature of the resistance to a few drugs has been identified as related to altered transport, modification of an enzyme, and a change in membrane composition (Lupetti et al., 2002). Additionally, antimicrobial peptides are promising candidates for the treatment of fungal infections since they have both mechanisms of action distinct from available antifungal agents and the ability to regulate the host immune defense systems as well (Lupetti et al., 2002). Probiotic strains that can be used for the treatment of vulvo-vaginal infection should be able to produce metabolites that are fungistatic for *C. albicans* and *C. glabrata* (Strus et al., 2005).

Though an umpteen number of peptide bacteriocins from *Enterococci* have been purified and genetically characterized over the last several years, yet the anti-*Candida* peptide/proteins are less investigated from the *E. faecalis* and *E. faecium*. Due to the increasing frequency of fungal infections in immunocompromised patients and development of an ominous trend in the treatment failures amongst the candidiasis patients receiving long-term antifungal chemotherapy resulted from the rapidly acquiring multidrug resistance (MDR) amongst pathogenic *C. albicans* a strong and pressing need has been felt in finding alternative form of antifungal antibiotics (Matejuk et al., 2010).

In this study, the APR210, the anti-*Candida* factor producer strain that was earlier identified as *E. faecalis* based on the biochemical tests and fatty acid methyl ester (FAME) analysis got redisgnated as *E. faecium* based on rDNA sequence analysis (Shekh et al., 2011), biochemical tests (e.g., L-Arabinose+, Raffinose+, Melibiose and Sorbitol−), and PCR-amplification data obtained using the *E. faecium*-specific primers (Cheng et al., 1997) and the genomic DNA of the producer strain. In this study the anti-*Candida* protein produced by the wild type *E. faecium* was gel-filtration purified and the peptides responsible for the anti-*Candida* activity in the pooled active fractions was identified by LC-ESI-MS/MS Supplementary 1 (Image 1-a gel picture).

## Results

We attempted to purify the antimicrobial protein by using crude proteins in the cell free supernatant. A three-step method was followed that included salting-out by ammonium sulfate fractionation, ion exchange and gel filtration chromatography. The fractions collected from each step of purification was checked for the antimicrobial activity by cut-well agar assay using three *C. albicans* strains that are different in drug-resistance pattern and are from different sources. The protein band that produced the anti-*Candida* activity in the zymogram assay was subjected to the LC-ESI-MS/MS analysis. The PCR-amplified product corresponding to the LysM domain gene was sequenced and analyzed.

### Gel filtration of the antimycotic protein

**Figure 1:**
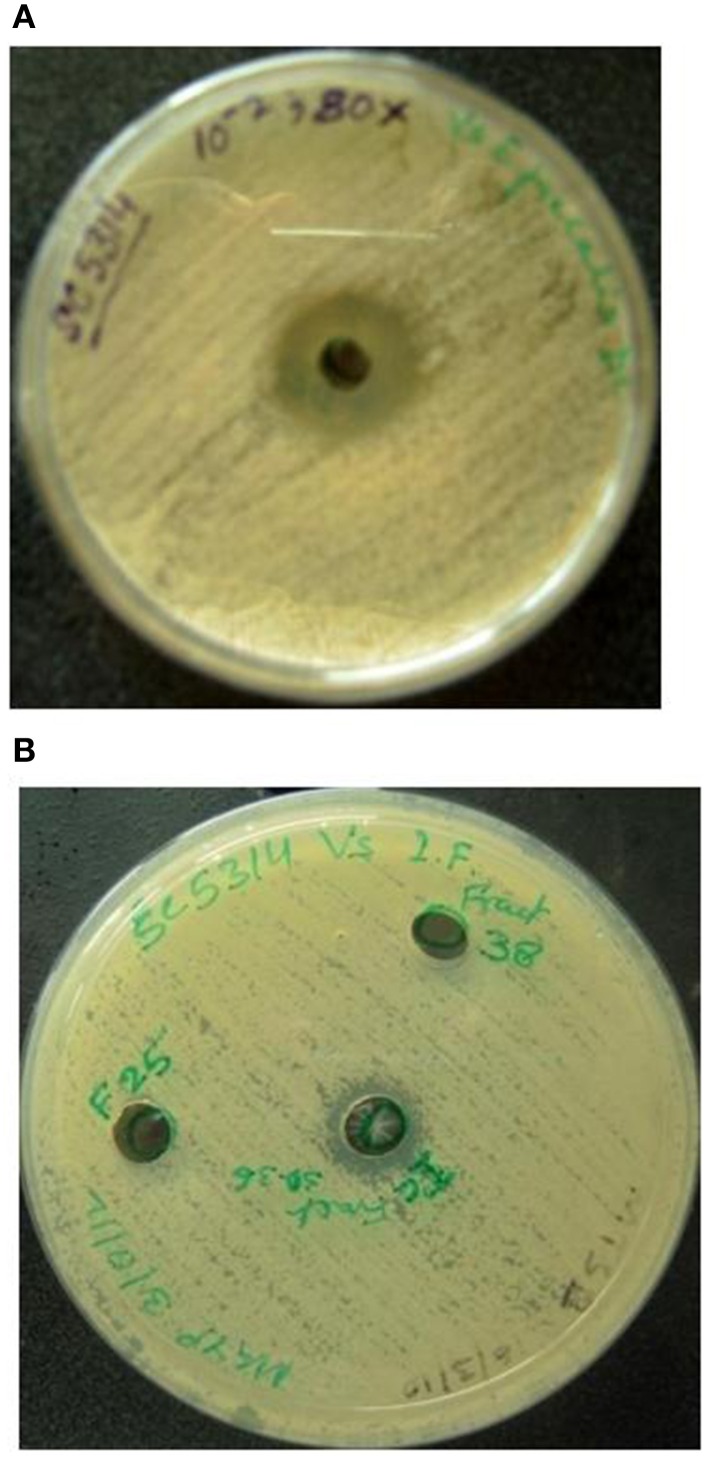
**(A)** Dialyzed concentrate (after ammonium sulfate fractionation and dialysis) shows clear zone of inhibition against *C. albicans* SC 5314. F25 and F38 denote ion-exchange fractions showing no inhibition. **(B)** Anti-*Candida* activity of pooled (30–36) fractions (collected during DEAE-Sepharose anion exchange chromatography) against *C. albicans* SC 5314.

**Figure 2:**
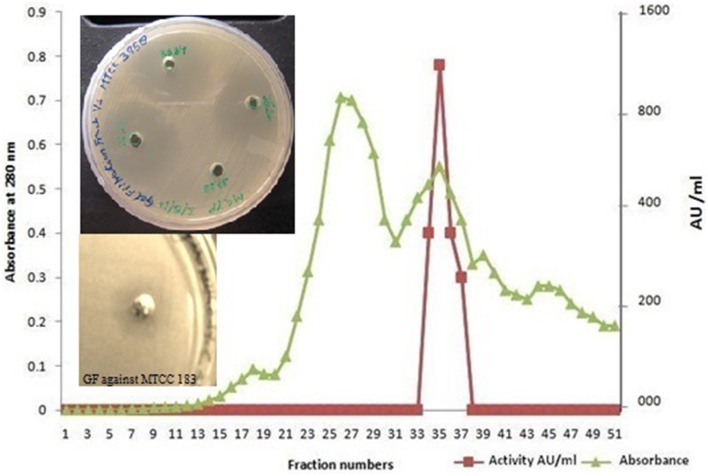
**Gel filtration elution profile of ACP on Sephadex G 75 column**. Anti-*Candida* activity was detected in fractions 31–36. The pooled fractions showed a distinct anti-*Candida* activity against *C. albicans* MTCC 3958 and MTCC 183 in the inset. ■, represents AU ml^−1^; ▴, represents absorbance at 280 nm.

**Figure 3:**
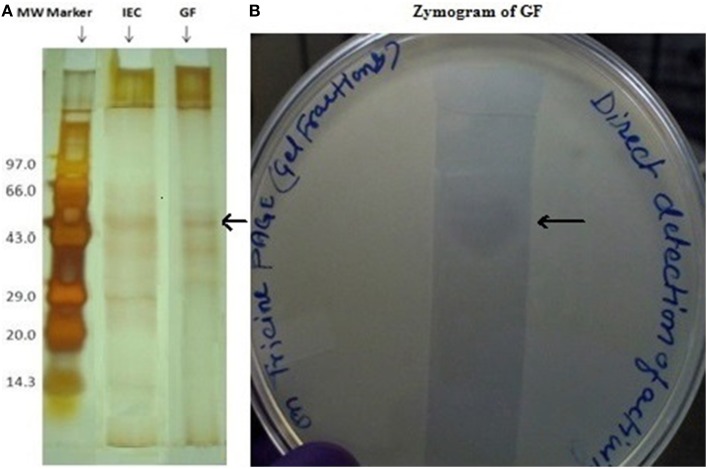
**(A)** Tricine SDS-PAGE (10%) profile of ion exchange and gel filtration fractions having biological activity in zymogram. Lane1 (from left) shows the molecular weight marker, lane 3 pooled ion exchange fractions (IEF) and lane 4 gel filtration fractions (GF). The arrow shows the band that showed in-gel inhibition in a zymogram assay. **(B)** Zymogram (right side) of the gel-filtration purified fraction after concentration.

Dialyzed concentrate (after ammonium sulfate fractionation and dialysis) showed a clear zone of inhibition against *C. albicans* SC 5314 (Figure 1A) and MTCC 3958 (photo not shown). Prior to gel filtration, the ion exchange fractions were tested against *C. albicans* MTCC 3958, 183, and SC 5314. The ion exchange fractions showed clearly the antimicrobial activity against these three yeast strains. However, we have shown here the activity against SC 5314 only (Figure 1B). The Sephadex G-75 gel filtration chromatography showed that the recovery of antifungal activity was approximately 22-fold (Shekh and Roy, 2012) with a reduction of 45%. The chromatogram of fractions collected during gel filtration on Sephadex G-75 is shown in Figure 2. The fractions were tested against *C. albicans* MTCC 3958, MTCC 183, and SC 5314. It was observed that fractions between 31 and 36 showed antimicrobial activity against MTCC 3958 and MTCC 183 (Figure 2 inset), whereas no zone of inhibition was recorded against SC 5314. These active fractions were pooled for further processing. As observed in Figure 3A, 10% SDS-PAGE fractionation indicated the presence of a protein band from ion exchange and gel filtration fractions that showed antimicrobial activity in the zymogram assay of Tricine Native PAGE (Figure 3B). Minimum inhibitory concentration (MIC) values against MTCC 183 and 3958 were 133 and 267 μg/ml respectively as determined in the earlier study (Shekh and Roy, 2012).

#### PCR-amplification from the N-terminal sequence

N-terminal amino acid analysis of the ion-exchange purified antimicrobial protein revealed the following partial sequence: NH2-DEVYTVKSGDSL (Shekh and Roy, 2012). Homology search was performed using NCBI BLAST (http://blast.ncbi.nlm.nih.gov/). The sequence DEVYTVKSGD displays high similarity (as evident from the *e*-values) to LysM domain containing protein of most of the *Enterococcus* species 29 amino acids residues away from the N-terminus of the LysM domain protein (sequences similarity and alignments are shown below). This sequence demonstrated identity with LysM domain proteins of six *E. faecium* strains.

**Figure 4:**
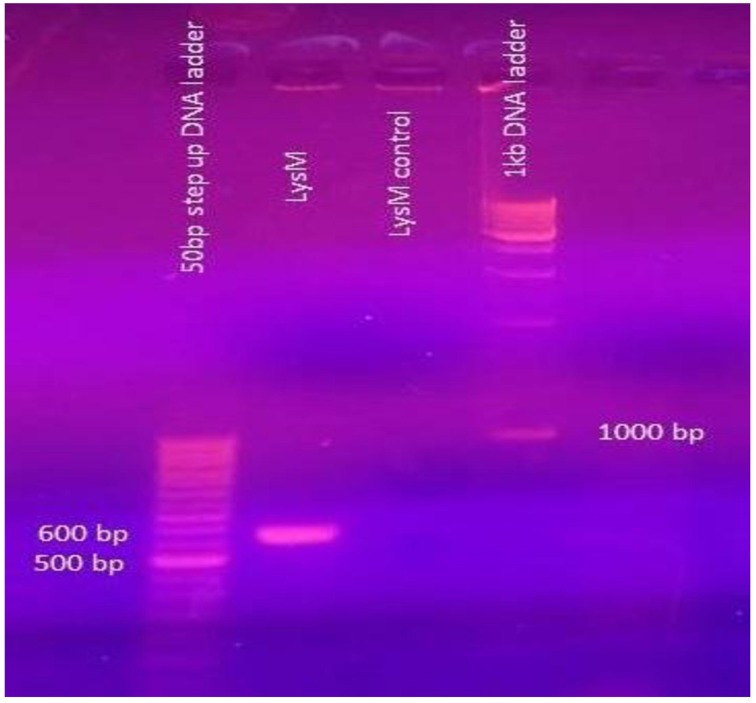
**From the left. Lane 1 50 bp step-up DNA ladder; Lane 2 574 bp amplified fragment of LysM gene; Lane 3**. Control reaction showing no amplification, Lane 4 1 kbp DNA ladder.

The forward (34–50) and reverse (592–610) primers were designed based on the N-terminal and C-terminal sequence of the LysM domain respectively. The 574 bp amplicon (Figure 4) was obtained by PCR and was further sequenced and the sequence analysis revealed 94.7% similarity with the 574 bp LysM domain-encoding gene of the strain *E. faecium* DO chosen for the PCR amplification. The nucleotide sequence that encodes 12 amino acids (DEVYTVKSGDSL) previously found as a result of N-terminal sequencing was detected.

### Identification of the protein by LC-ESI–MS/MS

**Figure 5:**
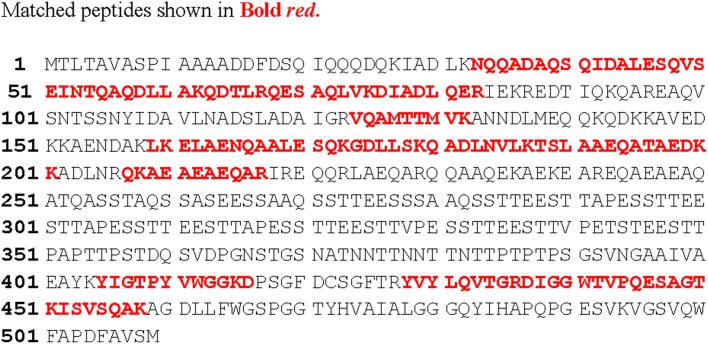
**Aligned amino acid sequence (in red, bold font) of the novel protein with sequences from the amino acid database of Mascot and LudwigNR**.

**Figure 6:**
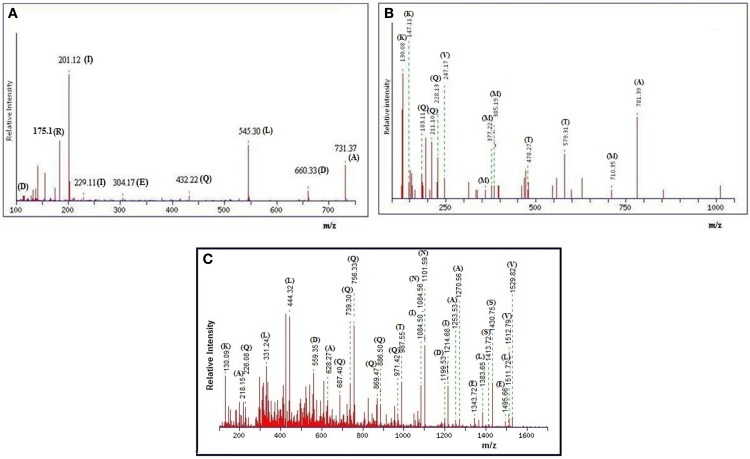
**(A)** MALDI-TOF spectra of anti-*Candida* peptide DIADLQER, produced by *E. faecium*. **(B)** MALDI-TOF spectra of anti-*Candida* peptide VQAMTTMVK, produced by *E. faecium*. **(C)** MALDI-TOF spectra of anti-*Candida* peptide NQQADAQSQIDALESQVSEINTQAQDLLAK, produced by *E. faecium*.

The gel-filtration purified band that showed anti-*Candida* activity in zymogram assay (Figure 3B, right side plate), was subjected to ESI-MS/MS analysis. The resulting protein sequence was blasted against the NCBI database with the best overall match showing 30 % identity with the NlpC/P60 family protein Tax_Id=791161 protein of *E. faecium* [PC4.1]. Out of 509 amino acids of the NlpC/P60, 149 amino acids were detected by LC-MS/MS and the subsequent data analysis revealed that 30% sequence match with the NlpC/P60 family protein (Figure 5). Based on the LC-ESI-MS/MS, the peaks corresponding to (NQQADAQSQIDALESQVSEINTQAQDLLAK (Figure 6C), DIADLQER, VQAMTTMVK, and TSLAAEQATAEDKK) were obtained in the mass spectra.

The peptide sequence NQQADAQSQIDALESQVSEINTQAQDLLAK revealed the score 96.0 with an expected value of 3.4e-^08^ in LudwigNR database, tr|D4VYT1|NlpC/P60 family protein Tax_Id=791161 [*E. faecium* PC4.1] whereas the peptide sequence TSLAAEQATAEDKK scored 69 (hall mark of identity) with an expected value of 5e-^05^. The individual peaks with corresponding amino acids are shown in Figures 6A–C.

### Protein view

MASCOT Search Results: Protein View: D4VYT1

tr|D4VYT1|NlpC/P60 family protein Tax_Id=791161 [*E. faecium* PC4.1]

**Table:** 

Database:	LudwigNR
Score:	251
Nominal mass (M_r_):	53958
Calculated pI:	4.35
Protein sequence coverage 30%.	

## Discussion

A number of species belonging to the genus *Enterococcus* have been reported to synthesize bacteriocins active against Gram-positive and negative bacteria. However, the reports on the anti-*Candida* activity of *Enterococcus* are rather rare.

In the present investigation, the anti-*Candida* substance was purified to a near homogeneity from the cell free supernatants via a three-step purification protocol. Sephadex G-75 gel filtration fractions containing nearly purified protein showed antimicrobial activity against *C. albican*s MTCC 3958 and MTCC 183 (Figure 2).

Based on the 12 amino acid residues from the N-terminal sequence determined in the previous study (Shekh and Roy, 2012), primers were designed and the PCR-amplified fragment of size 574 bp obtained (Figure 4) using the purified genomic DNA (as template) of the producer strain was sequenced; the nucleotide sequence was analyzed by BLAST using the NCBI search. The deduced amino acid sequence from the nucleotide sequence generated from the PCR amplified fragment (Figure 4) reveals the LysM motif that includes the DEVYTVKSGDSL. In a mutational analysis study conducted by Onaga and Tiara (2008), the LysM domain of PrChi-A was found to bind a chitin contributing significantly to the antifungal activity mediated by PrChi-A through their binding activity. The N-terminal sequence of PrChi-A was determined as DCTTYTVKSGDTCYAISQAN. This sequence is not homologous to the sequence of any plant chitinase, but very interestingly shares homology with the LysM of other proteins from several organisms (Onaga and Tiara, 2008). The nucleotide sequence encoding the LysM motif and the N-terminal sequence belonging to the LysM motif derived from the protein band that produced the zone of inhibition in zymogram (in-gel assay) in our study revealed striking similarity with the PrChi-A that exhibited antifungal activity (Onaga and Tiara, 2008). The shaded serine amino acid residue present adjacent to YTVKSGD is of same nature since threonine is the hydroxylated version of serine. However, the CYA amino acid residues in the N-terminal sequence of LysM of PrChi-A was not found in the translated sequence deriving from the nucleotide sequence of the PCR-amplified product; this CYA was also not found in the LysM motif sequence of *E. faecium* DO; however the pink shaded amino acid stretch ISQ matched with the LysM motif. LysM is a protein domain of about 45 amino acids found initially in several bacterial autolysin proteins (Joris et al., 1992). The domains are also known to bind N-acetylglucosamine (GlcNAc)-containing glycan molecules including peptidoglycan from several bacteria and chitin from fungi (Bateman and Bycroft, 2000; Buist et al., 2008; Iizasa et al., 2010; Petutschnig et al., 2010). In the plant kingdom, LysM domains are found in receptors for chitooligosaccharide and related compounds. The PrChi-A was reported to bind to chitin in the fungal cell wall mainly through LysM domains and then it degraded the chitin by hydrolytic action. This led to disruption of the fungal cell wall and fungal growth inhibition (Onaga and Tiara, 2008).

**Figure 7:**
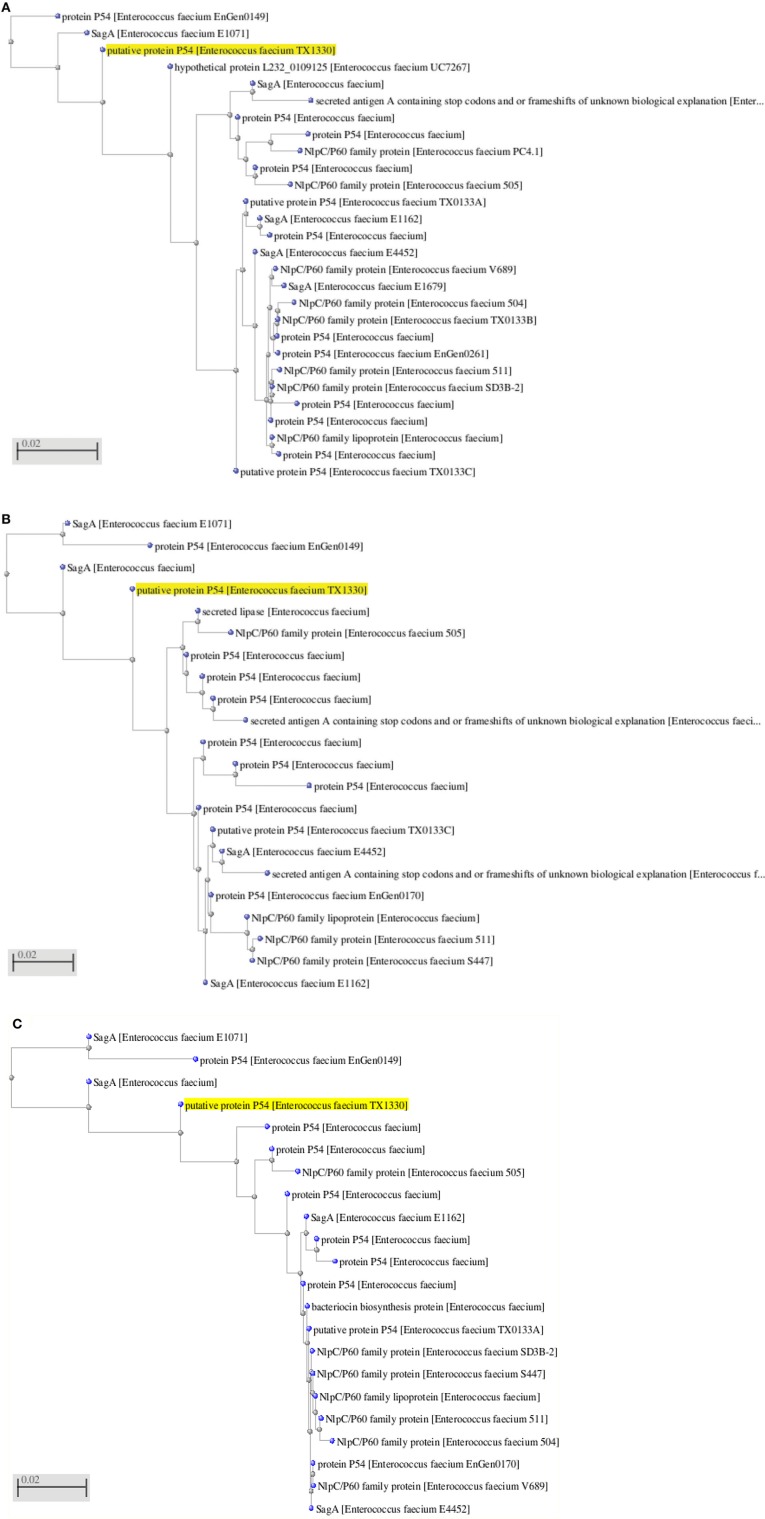
**(A)** Dendrogram presentation of the multiple alignment of the deduced amino acid sequence of NQQ. LAK of NlpC/P60 from Enterococcus sp. and other closely related strains. **(B)** Dendrogram presentation of the multiple alignment of the deduced amino acid sequence of VQAMTTMVK of NlpC/P60 from Enterococcus sp. and other closely related strains. **(C)** Dendrogram presentation of the multiple alignment of the deduced amino acid sequence of DIADLQER of NlpC/P60 from Enterococcus sp. and other closely related strains.

The LC-ESI-MS/MS generated the spectrum of separated peaks (Figures 6A–C) showing three abundant peaks, consistent with the molecular form of the purified protein. The resulting protein sequence was blasted against the NCBI database with the best overall match showing 30 % identity with the NIpC/P60 family (Anantharaman and Aravind, 2003). In mass spectrometry analysis, the protein sequence matched upto 30% with NlpC/P60 family protein present in the MASCOT database (Figures 6A–C, 7A–C). The Figures 7A–C, depict the dendrogram presentations of the multiple alignments of the deduced amino acid sequences of three peptides of NipC/P60 of *E.faecium* and other closely related strains. NlpC/P60 is a large family of cell-wall related cysteine peptidases that are broadly distributed in bacteria, archaea and eukaryotes (Anantharaman and Aravind, 2003). While their biochemical function seems to be conserved, the physiological roles of NlpC/P60 proteins are diverse, including cell separation, expansion, differentiation, cell-wall turnover, cell lysis, protein secretion and virus infection (Xu et al., 2010). Multiple proteins with NlpC/P60 domains are found in individual Gram-positive bacteria (Humann and Lenz, 2009). SagA of *E. faecium* belongs to this NIpC/P60 and is a secreted antigen which binds to the extracellular matrix proteins (Teng et al., 2003). Teng et al. (2003) reported the N-terminal sequencing of the fibrinogen- binding protein revealing a 20-amino-acid sequence, DFDSQIQQQDQKIADLKNQQ, identical to the predicted N-terminal sequence of the mature SagA (Teng et al., 2003). In our study, the significant peptides NQQADAQSQIDALESQVSEINTQAQDLLAK, DIADLQER, VQAMTTMVK, and TSLAAEQATAEDKK detected were matched with secreted antigen Sag A/SagBb proteins produced by the *E. faecium* strain. NlpC/P60 proteins are often fused to auxiliary domains, many of which are known cell-wall binding modules (e.g., LysM domain) (Xu et al., 2010). These auxiliary domains may be thought to function as targeting domains which localize their proteins to the cell wall (Xu et al., 2010). The species designation of *E. faecium* isolates was confirmed by amplification of specific DNA sequences by PCR. The results obtained in this study reveal a so far not described function for enterococcal LysM domain protein and taken together our findings clearly indicate the presence of this auxiliary domain in the form of LysM domain and NIpC/P60. However, the functional synergy between the NlpC/P60 domains and their auxiliary domains (Xu et al., 2010) needs to be investigated further to establish the rationale of our findings in the present study.

Extracellular *E. faecium* Sag A/Bb protein that is antigenic in nature is apparently essential for growth and shows broad-spectrum binding to extracellular matrix (ECM) proteins forming oligomers (Humann and Lenz, 2009) whereas the secreted protein sspA or sspB produced by *Streptoccoccus sp*. was reported to adhere to collagen type I and *C. albicans* (Teng et al., 2003). Insertion inactivation experiments have shown that both sspA and sspB genes are necessary for the binding of *S. gordonii* cells to *C. albicans* (Holmes et al., 1996). The SspB protein, when expressed on the surface of *E. faecalis*, confers upon the enterococcal cells the ability to bind *C. albicans* (Teng et al., 2003). In the same study conducted by Teng et al. (2003) the gene in clones d1–27 and d2–29 named Sag A for major secreted antigen was found in all 11 *E. faecium* strains from different communities and/or from different geographic sources (Teng et al., 2003).

The results obtained in the present work are in partial agreement with the earlier works. The identified peptide sequences derived from the LC-ESI-MS/MS match partly with the secreted antigen A/Bb that might impart binding ability toward the extracellular matrix present on the cell surface of *C. albicans* and the bacteriocin secretion accessory proteins might play an important role in the antimicrobial activity of the producer strain. The SagA (often called P60), P54 from *E. faecium* and SagBb secreted by *Enterococcus hirae* (Teng et al., unpublished data; Muller et al., 2006) have been reported to be associated with the cell wall biosynthesis; however these secreted proteins have the likelihood in mediating cell wall hydrolysis to indicate that apart from the binding to the cell surface of *C. albicans*, Sag A/Bb might play a role in antimicrobial activity indirectly.

## Methods

### Bacterial strains, growth conditions, and media

The producer test strain was routinely propagated in TGYE (tryptone, 5 g l^−1^; glucose, 1 g l^−1^; yeast extract, 3 g l^−1^, pH 7.2 ± 0.4) medium and was grown at 14 ± 0.5°C. The producer strain *E. faecium* and three indicator strains (*C. albicans* MTCC 3958, MTCC 183, and SC 5314) used in the present study were maintained in glycerol stock at −70°C (Shekh et al., 2011) and subcultured as and when required.

### Gel-filtration of ACP

The anti-*Candida* protein (ACP) was partially purified from supernatant of cultures of *E. faecium* using ammonium sulfate fractionation and ion exchange chromatography (Shekh and Roy, 2012) which was followed by gel filtration chromatography. The dialyzed sample was loaded onto a DEAE-Sepharose Fast flow column (GE Healthcare) equilibrated with 20 mmol sodium phosphate buffer, pH 8.0. The fractions were eluted using a linear gradient of 0–0.30 M NaCl (Shekh and Roy, 2012). The ion exchange fractions exhibiting the anti-*Candida* activity were pooled and further purified by gel permeation chromatography. Three grams of Sephadex G-75 were soaked in 200 ml of sterile distilled water and washed three times for removing fine particles, and then dissolved in 20 ml of sterile distilled water and poured in a 1 × 50 cm column. Void volume was determined by passing blue dextran (2000 kDa) through the column. The pooled ACP fractions (2.0 ml) were loaded onto the gel filtration column. The above mentioned buffer was used to elute the fractions each of 1.5 ml; those fractions were collected at a flow rate of 65 ml h^−1^ and read at 280 nm using UV-Visible spectrophotometer (Shimadzu). Antimicrobial assay for all fractions was performed against the freshly grown *C. albicans* MTCC 3958, MTCC 183 and SC 5314 by using cut-well agar assay (Shekh et al., 2011). The active fractions were concentrated by U-tube concentrator. That facilitated the removal of small molecules and also any salts present. The concentrated samples (100 μL) were added into a well of diameter 7.0 mm made in a freshly prepared MGYP agar plate which was seeded with freshly grown (diluted to 10^6^–10^7^ cells/ml) *C. albicans*. After 48 h of luxurious growth of *C. albicans* at 37°C, the plates were inspected for the zone of inhibition and the zone diameter was measured in terms of millimeter.

### Tricine SDS-PAGE

Samples were separated on a one dimensional SDS-PAGE. Slabs of 10% polyacrylamide (acrylamide/bisacrylamide, 30:0.8) with a 5% stacking gel were electrophoresed at 100 mV until the bromophenol dye reached the bottom of the gel. The gel was viewed after silver staining.

### Direct detection of biological activity on tricine PAGE

Tricine Native-PAGE (10%) (Schagger and Von Jagow, 1987) followed by a gel overlay was performed with active pooled fractions from gel filtration. After electrophoresis for 2 h at 20 mA, 2 duplicate gels were cut. One of the gels was silver stained. The other gel was fixed in 20% (v/v) isopropanol and 10% (v/v) acetic acid for 30 min, rinsed with 500 ml of MilliQ water for 1 h, and placed aseptically on an MGYP plate. To identify the active peptide band, the Tricine gel containing pooled active fraction was overlaid by freshly grown *C. albicans* MTCC 3958. After the agar solidified, the plate was incubated at 37°C for 48–72 h until *C. albicans* grew uniformly over the plate or an inhibition zone was observed.

### N-terminal amino acid sequence analyses

The protein band that showed inhibition in the in-gel assay in Tricine-Native PAGE was further subjected to N-terminal amino acid sequencing using an ABI Procise 494 protein sequencer (Applied Biosystems), Iowa State University, US (Shekh and Roy, 2012).

### PCR amplification and nucleotide sequencing

Based on the N-terminal sequence (Joris et al., 1992) of the LysM domain of the *E. faecium*, forward and reverse primers were designed. The primers used were: forward primer 5′ ACT TTT GCT GCT GGT GC 3′ and reverse primer 5′ TTA GTA CCA GCC GTT TGC 3′; a 25 μl of reaction consisted of 100 ng of purified genomic DNA of the producer strain, forward and reverse primers (30 picomoles each) and 200 μM of dNTPs each, and 1.5 units of Taq polymerase. The PCR conditions consisted of an initial denaturation step at 94°C for 90 s, followed by 35 cycles of denaturation 60 s at 94°C, annealing at 50 s at 48°C, and 50 s at 72°C. The final extension step was at 72°C for 3 min. The nucleotide sequence was determined on both strands by using Big Dye Terminator chemistry and ABI 3500 × L Genetic Analyzer. The sequence data was evaluated on the basis of sequence homology to GenBank entities using BLASTN and was analyzed using the open reading frame finder of NCBI.

### Mass spectrometry of the gel-filtration purified protein

The gel filtration purified fractions that contained anti-*Candida* principle were pooled and resolved using the SDS-PAGE. The band in the stained gel was precisely cut and used for LC-ESI-MS/MS (Proteomics International, Australia). Protein samples were trypsin digested and peptides extracted according to standard techniques (Xu et al., 2010). Peptides were analyzed by electrospray ionization mass spectrometry using the Ultimate 3000 nano HPLC system [Dionex] coupled to a 4000 Q TRAP mass spectrometer [Applied Biosystems]. Tryptic peptides were loaded onto a C18 PepMap100, 3 μm [LC Packings] and separated with a linear gradient of water/acetonitrile/0.1% formic acid (v/v). Spectra were analyzed to identify proteins of interest using Mascot sequence matching software [Matrix Science] with Ludwig NR database. Spectra were obtained for the major peptide ions in MS mode and sequence data obtained when the spectrometers automatically reverted to MS/MS mode. These spectra were then compared with databases (MASCOT and LudwigNR) to provide hits that could identify matching or similar sequences. The amino acid sequences were identified using the tool provided by the National Center for Biotechnology Information (NCBI) and ORF Finder tool (www.bioinformatics.org/sms/orf=find.html) for the sequences obtained. The translated ORFs were compared to known sequences deposited in the non-redundant protein databases (www.ncbi.nlm.nih.gov) using the BLAST program (Shekh and Roy, 2012). Multiple alignments were performed with the CLUSTAL W proGram (Altschul et al., 1990) Supplementary Material Images 2–4.

### Conflict of interest statement

The authors declare that the research was conducted in the absence of any commercial or financial relationships that could be construed as a potential conflict of interest.
