# Supplementary material for: The anti-Candida activity by Ancillary Proteins of an Enterococcus faecium strain
Source: Front Microbiol. 2015 May 8;6:339. doi: 10.3389/fmicb.2015.00339 (PMC4424852; doi:10.3389/fmicb.2015.00339)
Supplement: Supplementary file 1 [file Image1.PDF]

The species identification of the producer strain *Enterococcus faecium* isolate was confirmed by amplification of specific DNA sequence by PCR using the set of primers given below (Cheng et al.1997).

A pair of 21-mer primers (EM1A, 5’-TTG AGG CAG ACC AGA TTG ACG-3’; EM1B, 5’-TAT GAC AGC GAC TCC GAT TCC-3’) was synthesized by IDT Technologies. 1.0 U of Taq DNA polymerase (Merck-Millipore), 10 and 100 ng of DNA templates and 30 and 50 picomoles of primers were used respectively. The amplification was accomplished by initial denaturation at 94 °C for 2 min, followed by 30 cycles of 45 s at 94 °C, 45 s at 55 °C and 1 min at 72 °C, in a PCR System thermocycler (Applied Biosystems). An extension step of 7 min at 72 °C was included after the final cycle. The PCR products were analyzed by electrophoresis on a 1.2% agarose gel. The PCR product was estimated of about 658 base pairs of amplicon size.

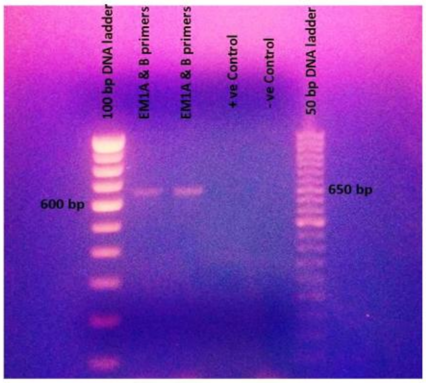

Agarose gel electrophoresis view for verification of the strain *E.faecium* showing molecular size of the amplicon of the expected size, 658 bp.
