# Supplementary figures and images for: The anti-Candida activity by Ancillary Proteins of an Enterococcus faecium strain
Source: Front Microbiol. 2015 May 8;6:339. doi: 10.3389/fmicb.2015.00339 (PMC4424852; doi:10.3389/fmicb.2015.00339)

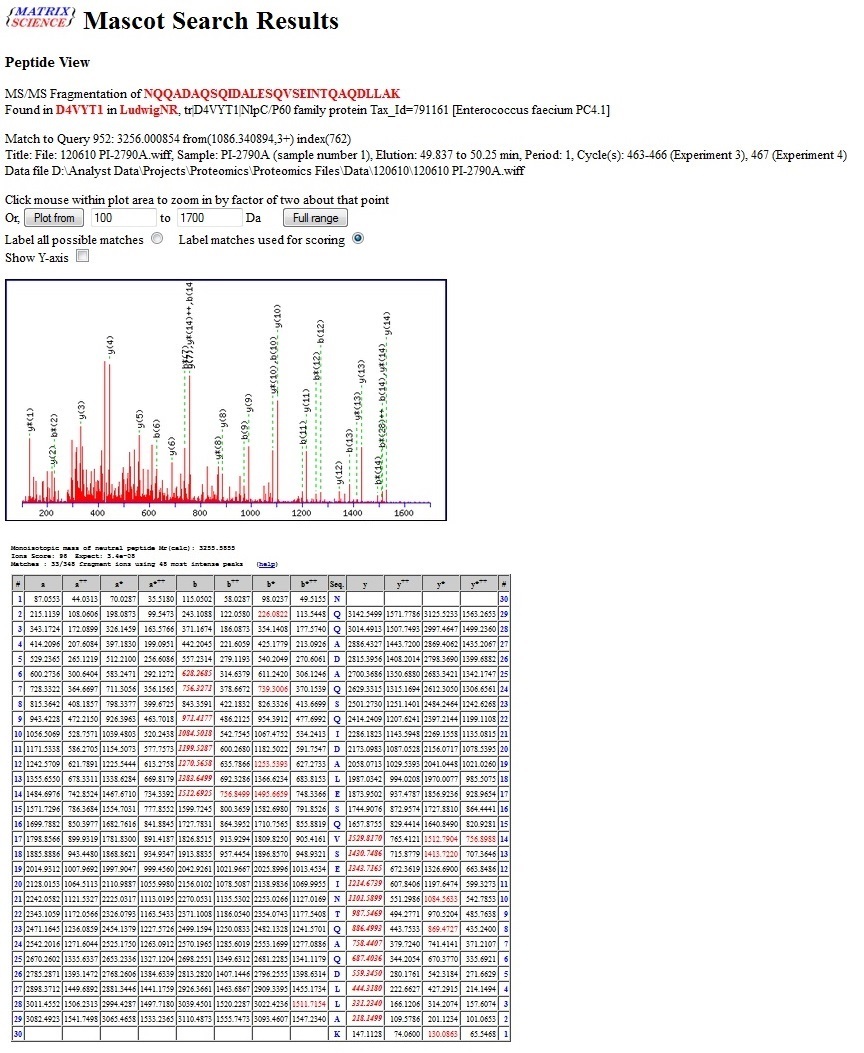

Supplement: Supplementary file 2 [file Image2.JPEG]

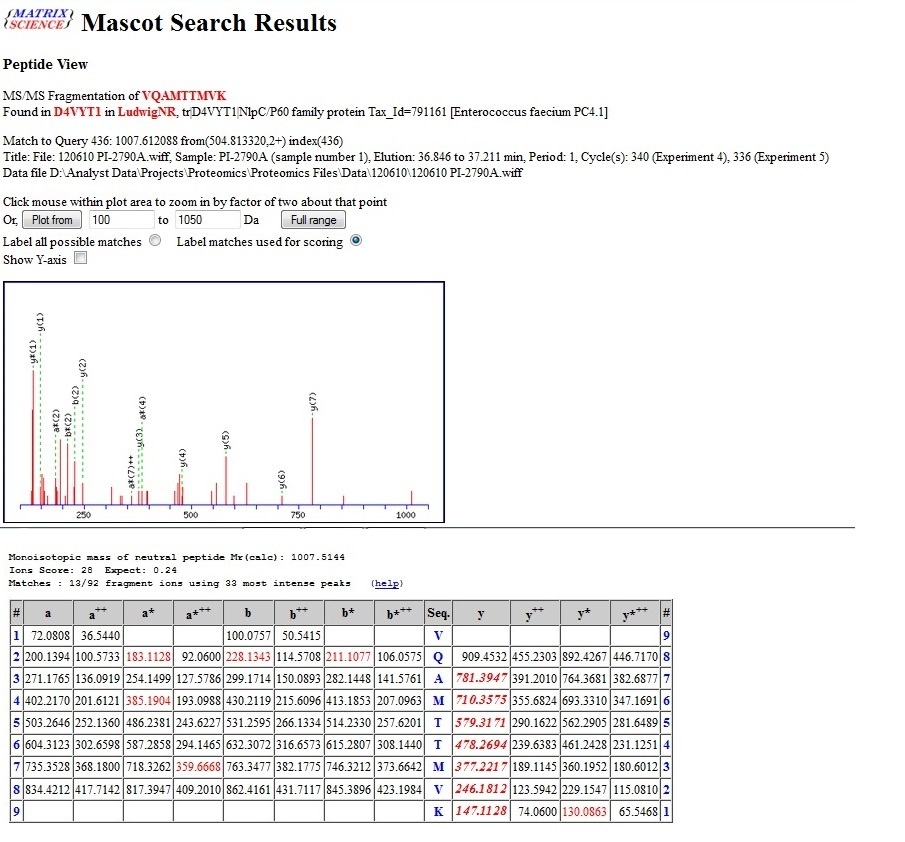

Supplement: Supplementary file 3 [file Image3.JPEG]

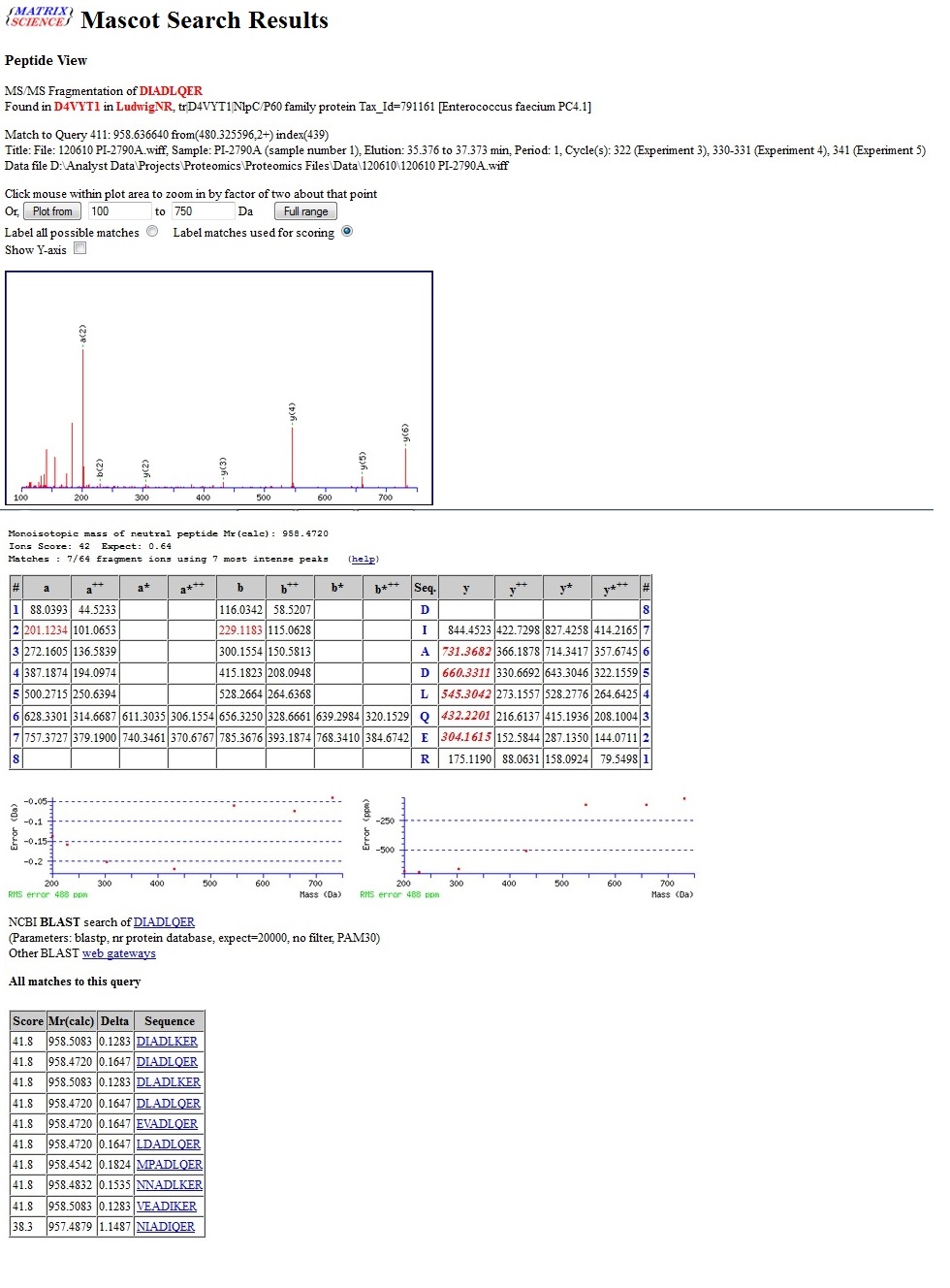

Supplement: Supplementary file 4 [file Image4.JPEG]
